# A microfluidic device based on an evaporation-driven micropump

**DOI:** 10.1007/s10544-015-9948-7

**Published:** 2015-03-25

**Authors:** Chuan Nie, Arjan J. H. Frijns, Rajesh Mandamparambil, Jaap M. J. den Toonder

**Affiliations:** 1 Department of Mechanical Engineering, Eindhoven University of Technology, P.O. Box 513, 5600 MB Eindhoven, The Netherlands; 2 Holst Centre, P.O. Box 8550, 5605 KN Eindhoven, The Netherlands; 3 Institute for Complex Molecular Systems, Eindhoven University of Technology, P.O. Box 513, 5600 MB Eindhoven, The Netherlands

**Keywords:** Evaporation, Flexible system, Flow rate control, Micropump

## Abstract

In this paper we introduce a microfluidic device ultimately to be applied as a wearable sweat sensor. We show proof-of-principle of the microfluidic functions of the device, namely fluid collection and continuous fluid flow pumping. A filter-paper based layer, that eventually will form the interface between the device and the skin, is used to collect the fluid (e.g., sweat) and enter this into the microfluidic device. A controllable evaporation driven pump is used to drive a continuous fluid flow through a microfluidic channel and over a sensing area. The key element of the pump is a micro-porous membrane mounted at the channel outlet, such that a pore array with a regular hexagonal arrangement is realized through which the fluid evaporates, which drives the flow within the channel. The system is completely fabricated on flexible polyethylene terephthalate (PET) foils, which can be the backbone material for flexible electronics applications, such that it is compatible with volume production approaches like Roll-to-Roll technology. The evaporation rate can be controlled by varying the outlet geometry and the temperature. The generated flows are analyzed experimentally using Particle Tracking Velocimetry (PTV). Typical results show that with 1 to 61 pores (diameter = 250 μm, pitch = 500 μm) flow rates of 7.3 × 10^-3^ to 1.2 × 10^-1^ μL/min are achieved. When the surface temperature is increased by 9.4 °C, the flow rate is increased by 130 %. The results are theoretically analyzed using an evaporation model that includes an evaporation correction factor. The theoretical and experimental results are in good agreement.

**Electronic supplementary material:**

The online version of this article (doi:10.1007/s10544-015-9948-7) contains supplementary material, which is available to authorized users.

## Introduction

For continuous sweat sensing and monitoring, a compact wearable device that can be worn directly on the skin and that continuously collects and analyzes the sweat would be an ideal solution (Brongersma et al. 2014; Curto et al. 2012; Schazmann et al. 2010). This requires a microfluidic device that not only automatically absorbs the sweat, but also an integrated micropump that generates a continuous flow along sensors located within the device.

Micro-pumps have been realized by a number of methods: by using piezoelectricity (Hsu and Sheen 2009; Hwang et al. 2008; Jang and Kan 2007; van Lintel et al. 1988), magnetohydrodynamics (Homsy et al. 2005; Lemoff and Lee 2000; Nguyen and Kassegne 2008), electrokinetics (Lastochkin et al. 2004), or electrohydrodynamics (Fuhr et al. 1992), These approaches introduce complexity in the system, are relatively expensive to integrate, and require a control loop to regulate the flow. Surface tension, or capillarity, can be used to create microfluidic flows using simpler systems (Berthier and Beebe 2007; Yun et al. 2002). However it is difficult to realize a continuous flow for a long duration using surface tension alone. By using the combination of evaporation and capillarity, micro-pumps can be realized that in principle run passively for a long time and that can be realized at reduced production cost and with simple processes and materials. Moreover, with heat introduced into the system, the flow rate can be adjusted by controlling temperature. The idea of using evaporation as the driving effect is inspired by the water cycle in plants: water is absorbed at the root of the plant, transported via the stem to the leaves, and evaporated through stomas on the leaf surface. In contrast to purely capillary driven pumps, for which the driving force stops when a fluidic system is completely filled, an evaporation driven micro-pump can generate a continuous and prolonged flow. This makes this approach appealing for application in sweat sensing and monitoring, for which a continuous flow over the sensor, e.g., an electrochemical sensor, is desired (Zevenbergen et al. 2011). Previously proposed wearable sweat sensors by Curto et al. (2012) and Schazmann et al. (2010) work by pure capillarity, on the basis of a microfluidic device with integrated absorbent material and a cotton thread integrated in a microchannel. Our pumping mechanism, in addition to capillarity, uses evaporation to drive the flow which offers an additional flow control mechanism.

Several publications describe the combined use of capillarity and evaporation to realize micropumps (Chen et al. 2012; Effenhauser et al. 2002; Goedecke et al. 2002; Juncker et al. 2002; Walker and Beebe 2002; Xu et al. 2008; Zimmermann et al. 2005). Various control methods (Table 1) are mentioned in these previous studies, namely changing temperature (Chen et al. 2012; Zimmermann et al. 2005), applying forced convection (Goedecke et al. 2002) and modifying the geometry (Chen et al. 2012). Flow rates achieved are in the range between 1.8 × 10^-3^ μL/min for passive pumping to 13.33 μL/min for actively controlled evaporation. Different materials were chosen in previous studies. Most of the reported water based evaporation driven pumps are non-flexible, are fabricated with cleanroom processes (Goedecke et al. 2002; Juncker et al. 2002; Xu et al. 2008; Zimmermann et al. 2005) i.e., silicon-wafer technology, using polydimethylsiloxane (PDMS) (Chen et al. 2012), or using membranes with a sorption agent (Effenhauser et al. 2002). However, for applications like ours, namely disposable and wearable sweat sensors, the pump should be cheap in fabrication, as well as flexible to ensure a good contact between the system and the skin and predictable in the pumping rate.

**Table 1:** Previous studies of water evaporation driven pumps

Authors	Base material	Method	Flow rate
Zimmermann et al.(2005)	Si	Active, heater	1.8 × 10^-3^ - 7.2 × 10^-2^ μL/min
Chen et al.(2012)	PDMS based	Active, heater (A) and Passive (P)	0.53-0.69 μL/min (P) 6.67-13.33 μL/min (A)
Xu et al.(2008)	Glass	Active, heater, RH control	0.54-2.34 μL/min
Juncker et al.(2002)	Si	Active, heater and Passive	4.8 × 10^-2^ μL/min (P), 1.2 μL/min(A)
Goedecke et al.(2002)	Glass	Active (air flow) and Passive	Maximum velocity: 2.25 mm/s
Effenhauser et al.(2002)	Through a membrane in a chamber	Active,(heater and sorption agent) and passive	0.01-1 μL/min

In this work, we introduce a flexible and controllable evaporation driven pump that can be used to drive a continuous fluid flow through a microfluidic-channel and over a sensing area. The key element of the pump is a micro-porous membrane mounted at the channel outlet, such that a pore array with a regular hexagonal arrangement is realized through which the fluid evaporates, which drives the flow within the channel. The system is completely fabricated on flexible polyethylene terephthalate (PET) foils, which can be the backbone material for flexible electronics applications, such that it is compatible with volume production approaches like Roll-to-Roll technology (van den Brand et al. 2008) to reduce the cost of the product both from material and manufacture aspects when compared to cleanroom processes. The flexibility of the device ensures compatibility with skin sweat sensing applications. The generated flows are analyzed experimentally using Particle Tracking Velocimetry (PTV). The evaporation rate from the outlet is analyzed using a model based on evaporation theory that includes evaporation correction factors. Experimental and theoretical results are compared directly. The possibility to design an evaporative micropump that can generate sufficient pumping rate for application as a wearable sweat sensor is discussed.

In addition, we demonstrate a simple and robust method to collect fluid through a filter-paper based interface layer between the device and the skin surface.

## Experiment

**Fig. 1:**
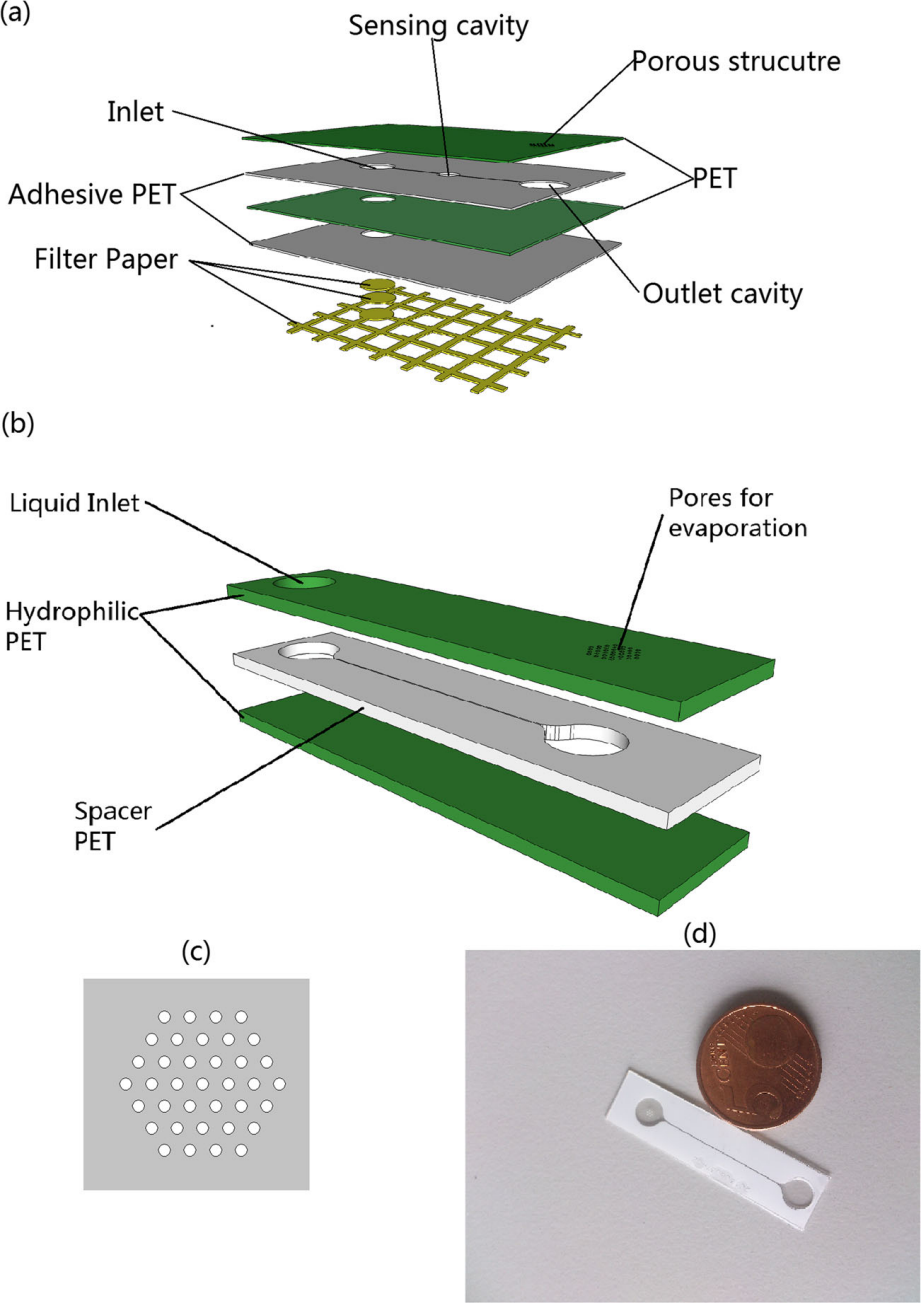
(**a**) Schematic of our proposed device, with a filter paper base for fluid collection, a microchannel that connects the inlet to a sensing cavity and then to the channel outlet cavity that is covered by a porous structure through which the fluid evaporates. (**b**) Simplified device, without filter paper and sensing cavity, which was used to quantitatively characterize the evaporative pumping behavior. (**c**) Schematic of the pore array at the evaporation end. (**d**) Real device that was used for flow rate characterization, compared with a 5 Eurocent coin

A schematic of our proposed microfluidic device is shown in Fig. 1a. Green layers in Fig. 1a are PET foils coated on both sides by an adhesive film, allowing for straightforward lamination. Filter paper (VWR 313, cut by CO_2_ laser VLS 3.5, Universal Laser Systems, yellow in Fig. 1a) is stuck onto the backside of the adhesive PET layer stack (white in Fig. 1a). The filter paper, cut in a square grid of lines with a width and spacing of 4 mm and 1 mm respectively, collects sweat from the skin surface, and transports the liquid to the inlet cavity of the microchannel. Two pieces of circular paper are used to fill the inlet cavity to ensure full contact between the liquid and the channel wall. The inlet cavity is connected to a sensing cavity and then to the outlet by microchannels. The sensing cavity can contain for example an electrochemical sensor, which will be integrated in future studies – here we focus on the microfluidics aspects.

To be able to quantitatively characterize the pumping behavior of the evaporation driven pump, we first used a slightly simplified device without filter paper and sensing cavity as shown in Fig. 1b. This device is made of three layers of flexible PET foils. The device has dimensions of 40 mm × 10 mm × 300 μm. The top and bottom layers are made of single side hydrophilized PET foil (3 M-9984) with a thickness of 100 μm. The middle layer (i.e., the spacer layer in Fig. 1b) is double side adhesive coated PET foil (TESA-IVD 09448PV1.001/09) with a thickness of 100 μm including adhesives. All structures are fabricated by laser processing. The channel structure (middle layer) with a length of 21.4 mm and the inlet (top layer) with a diameter of 5 mm are ablated by a CO_2_ laser (Universal VLS 3.5). The dimensions of the channel are 150–250 μm in width and 93.1 ± 3.6 μm in height, after the lamination process. The pore structure is manufactured by an excimer laser (Optec SA). The device is assembled by lamination using a rubber roller. A hexagonally distributed porous structure (Fig. 1c) is present at the outlet to realize an array of micropores that, by evaporation through the individual pores, drive the flow. The pores have a diameter ranging from 50 μm to 250 μm for different experiments and the matrix of pores has a fixed pitch of 500 μm.

When water is introduced at the inlet, it will be driven through the channel by a capillary force in the channel towards the outlet. Then the water gets evaporated through the porous structure and since the liquid meniscuses are pinned in the pores, this leads to a continuous flow in the channel from the inlet to the outlet. If applied as a wearable sweat sensor, the evaporation rate can be enhanced by the temperature of the skin, and it can be further enhanced and controlled with an active heater. The relationship between the surface temperature and the flow rate will be discussed later in this paper. During the pumping experiments, the inlet of our simplified device may dry out due to the evaporation at both outlet and inlet, however in the experiments, water is added at the inlet to avoid this effect, while keeping the inlet meniscus as flat as possible to minimize the pressure caused by the surface tension difference between the in- and outlet so that this effect does not influence the flow in the channel.

For the complete wearable sweat sensing device shown in Fig. 1a, the opening of the inlet is at the opposite side of the foil, facing the skin. Hence the influence of evaporation at the inlet will be absent. The inlet is in this case attached through a special filter paper interface and in that case the liquid is absorbed by the bottom filter paper layer and transported to the inlet cavity. During operation, even if the filter paper is completely filled, the evaporation will continue to drive the flow, provided the liquid is continuously supplied to the filter paper, i.e., at the skin’s surface, at a rate that is at least equal to the evaporation rate.

The flow velocity in the channel is measured by adding density matched tracer particles (R0200B, diameter 2 μm, from Thermo Scientific) to the liquid. The displacements of the tracer particles with respect to the flow in the channel are recorded by a camera (Altra 20) mounted on an optical microscope (Olympus BX 51). The position of the mid-plane is found on the scale of the focus knob of the microscope, after focusing on the top and bottom surface of the channel. For every single experiment, 10 recordings having a length of one minute are taken, at a frame rate of 10 to 20 Hz. The environmental conditions are monitored by a humidity and temperature (RH/T) sensor (IST HYT-271). In the passive evaporation experiments without any heater, we assume that the liquid temperature is equal to the air temperature.

**Fig. 2:**
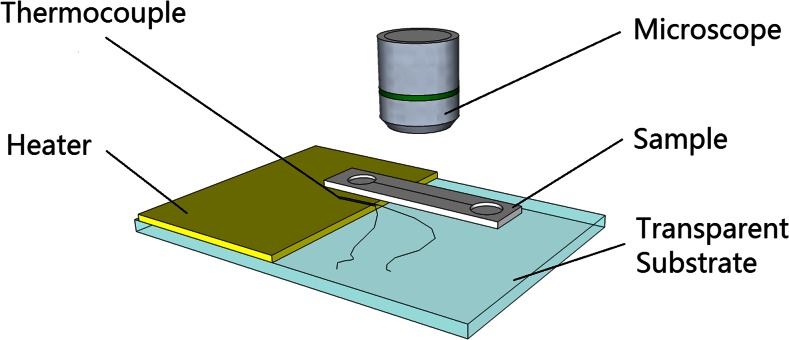
Experimental setup for an evaporation driven flow with a heater: The evaporation end of the sample is placed on the heater and the surface temperature rise of the heater is recorded by the thermocouple

The evaporation of water in air is enhanced by heating the outlet. A series of experiments is run on a voltage controlled heater (PTC heater, maximum 12 V 3 W from thermo technologies) to investigate the influence of temperature on the evaporation process because of the on-skin applications. The evaporation end of the simplified device is put on the heater to achieve a temperature rise, as shown in Fig. 2. A J-type thermocouple is attached to the heater surface close to the evaporation outlet to record the temperature rise of the heater. The heater is connected to a voltage source to control the power and therefore the temperature on the surface. In the set-up shown in Fig. 2, the heater is not mounted underneath the inlet port in order to prevent extra evaporation at the inlet from influencing the measurement. In the complete device that represents the sweat sensor device (Fig. 1a) the upward facing inlet port is absent, so this additional evaporation is of no concern and the entire device would be heated when attached to the body’s surface.

The recorded videos are post-processed by a computer using a two-dimensional Particle Tracking Velocimetry (2D-PTV) method. The traveled distances of the particles are calculated using MATLAB (V2010b). After plotting the velocity distribution over the channel width, analytical curve fitting is performed and the flow rate in the channel is calculated, as will be described in more detail later.

## Theory

In our device, the evaporation takes place through the individual pores of the porous membrane at the outlet, as shown in Fig. 1b. This leads to a total volumetric liquid evaporation rate of ${\left(\frac{\mathbf{dV}}{\mathbf{dt}}\right)}_{\mathbf{e}}$ which drives a volumetric flow rate **Q** through the cross-section of the channel. By definition, these two must be in equilibrium:

1 $$ {\left(\frac{\mathbf{dV}}{\mathbf{dt}}\right)}_{\mathbf{e}}=-\mathbf{Q} $$

The evaporation rate ${\left(\frac{\mathbf{dV}}{\mathbf{dt}}\right)}_{\mathbf{e}}$ can be estimated by the theoretical considerations explained below, and the internal volumetric flow rate **Q** can be determined experimentally by using velocimetry methods, for example Particle Tracking Velocimetry (PTV) as will be explained below as well.

### Evaporation rate

**Fig. 3:**
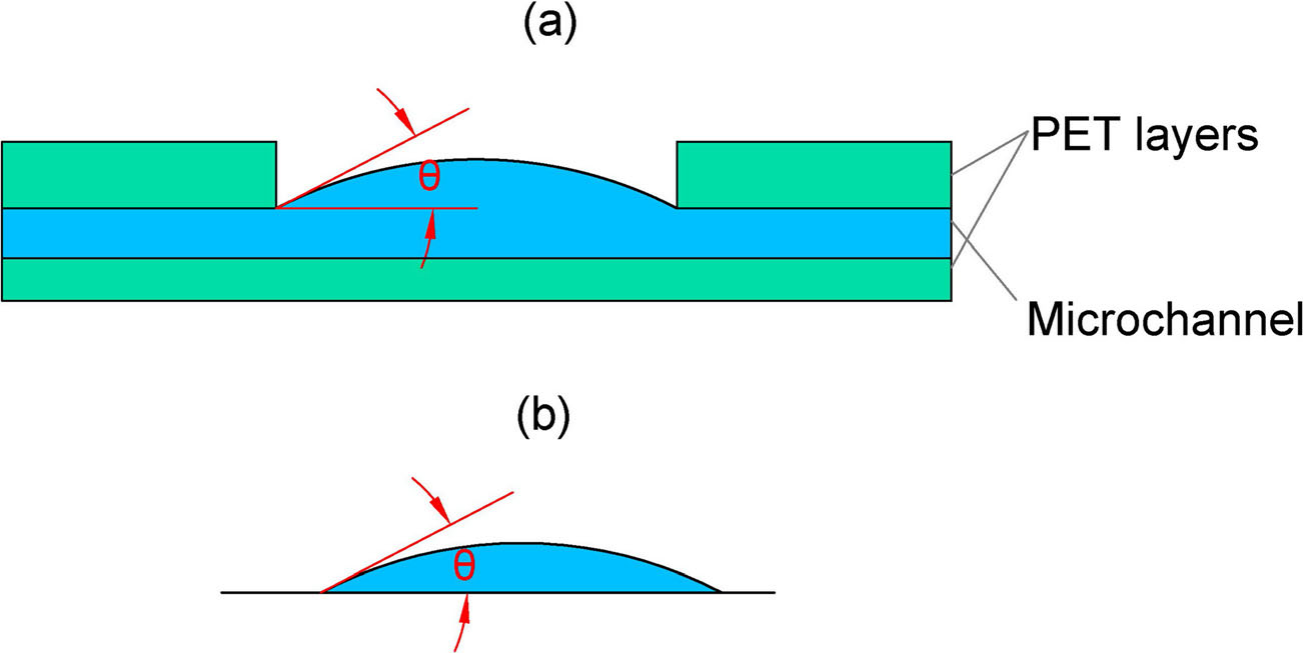
(**a**) the geometry of the liquid surface within a single pore of our microporous membrane. The shape of the surface will be addressed below. (**b**) The approximate case of a sessile droplet on a flat surface. The evaporation through the pore in (**a**) can be approximately described by a model developed for the evaporation of a sessile droplet (**b**), including the case of a flat liquid surface (i.e., θ = 0)

To estimate the evaporation rate, we first consider the evaporation through a single pore. The configuration is shown in Fig. 2a: the liquid is pinned at the edges of the pore and forms a circular surface with curvature defined by the angle *θ*. This case can be approximately described by the theory presented by Picknett and Bexon (Picknett and Bexon 1977). They present a model that describes evaporation from a surface bounded by a circle (which can be curved like a lens or a drop) based on solving the diffusion equation, through an analogy with electrostatic potential theory. This theory has been used to describe the evaporation of sessile drops, as shown in Fig. 2b, which is similar. The resulting equation reads (see also (Semenov et al. 2011)):

2 $$ {\left(\frac{\mathbf{dV}}{\mathbf{dt}}\right)}_{\mathbf{single}}=-2\frac{\pi \mathbf{D}\mathbf{M}}{\rho}\left[\mathbf{c}\left({\mathbf{T}}_{\mathbf{w}}\right)-\mathbf{H}\mathbf{c}\left({\mathbf{T}}_{\mathbf{a}}\right)\right]\mathbf{F}\left(\theta \right)\mathbf{a} $$

where D is the diffusivity of vapor at ambient temperature, **a** is the radius of the bounding circle (i.e., the drop radius in case of sessile drops), M is the molecular mass of the vapor molecules, *ρ* is the density of the liquid. **c**(**T**_**w**_) and **c**(**T**_**a**_) are the saturated water molar vapor concentration in air at the liquid–gas interface and ambience, respectively. **c**(**T**_**w**_) and **c**(**T**_**a**_) depend sensitively on the temperature, and approximate values are given in the appendix. **H** is the relative humidity at the ambience. **F**(*θ*) is a function of the contact angle *θ* indicated in Fig. 3, which is given by (Picknett and Bexon 1977; Schönfeld et al. 2008).

3 $$ F\left(\theta \right)\left\{\begin{array}{c}\hfill 2/\pi, \kern1.35em \mathbf{f}\mathbf{o}\mathbf{r}\ \theta =0\hfill \\ {}\hfill \frac{0.6366\theta +0.09591{\theta}^2-0.06144{\theta}^3}{ \sin \theta },\kern0.6em \mathbf{f}\mathbf{o}\mathbf{r}\ 0<\theta \le 0.175\hfill \\ {}\hfill \begin{array}{l}\Big(0.00008957+0.6333\theta +0.166{\theta}^2-0.08878{\theta}^3\\ {}+0.01033{\theta}^4\Big)/ \sin \theta, \kern0.97em \mathbf{f}\mathbf{o}\mathbf{r}\ 0.175\le \theta \le \pi \end{array}\hfill \end{array}\right. $$

Note that equations (2) and (3) describe the evaporation rate from a single flat or curved surface bounded by a circle, with contact angle *θ* in the complete range between *θ* = 0 (i.e., a flat circular film on a plane) to *θ* = *π* (i.e., a spherical drop on a plane). The assumptions of the theory are that the evaporation takes place by pure diffusion, that the environment is still air, that the temperature is uniform, that gravity does not play a role, that no liquid flow takes place within the droplets, and that the substrate is flat and smooth. In our experiments, conditions are controlled such that the first four assumptions hold, however the latter two assumptions are valid only approximately: the vertical walls of the pore may play a role (see Fig. 3a), and flow is occurring by definition since we use the evaporation to drive a flow. If (1) the pore is wide compared to its height and (2) the local flow is small, the expected deviations are small.

Compared to an isolated single pore, the evaporation through an array of multiple droplets, like we have in our microporous membrane, is subject to interpore interaction and is reduced by a local vapor field created by the neighboring pores. To account for this, we use, by analogy, an evaporation correction factor (*η*) that has been introduced to account for the interaction between evaporating drops on a surface. This factor depends on the geometry of the droplet array, and can be obtained through computationally demanding calculations (Imaoka and Sirignano 2005). Here, we use an approximate model introduced by (Annamalai et al. 1993), for which good agreement with experiments was found when the ratio between the radius of the droplet and the pitch of the array is small: in a rectangular droplet array, the error of evaporation rate was estimated to be less than 10 % when the pitch to radius ratio is larger than four. According to this model, in the case of an array of N pores, the evaporation rate through the i^th^ pore can be described by multiplying the evaporation rate through a single pore by the evaporation correction factor (*η*_**i**_) (see also (Kokalj et al. 2010) who describe arrayed-droplets evaporation for cooling applications):

4 $$ {\left(\frac{\mathbf{dV}}{\mathbf{dt}}\right)}_{\mathbf{i}}={\eta}_{\mathbf{i}}{\left(\frac{\mathbf{dV}}{\mathbf{dt}}\right)}_{\mathbf{single}} $$

The evaporation correction factor through the i^th^ pore (*η*_**i**_) in the array follows the linear equation:

5 $$ {\eta}_{\mathbf{i}}+{\displaystyle \sum_{\mathbf{j}=1,\mathbf{j}\ne \mathbf{i}}^{\mathbf{N}}}\left({\eta}_{\mathbf{i}}\frac{{\mathbf{a}}_{\mathbf{i}}}{\left|{\mathbf{r}}_{\mathbf{i}}-{\mathbf{r}}_{\mathbf{j}}\right|}\right)=1 $$

where **a**_**i**_ is the radius of the i^th^ pore and |**r**_**i**_ − **r**_**j**_| is the distance between the i^th^ and j^th^ pore. In a uniform pore array of total N droplets, the mean evaporation correction factor is (Kokalj et al. 2010)

6 $$ \eta =\frac{1}{\mathbf{N}}{\displaystyle \sum_{\mathbf{i}=1}^{\mathbf{N}}}{\eta}_{\mathbf{i}} $$

and the total evaporation rate is

7 $$ {\left(\frac{\mathbf{dV}}{\mathbf{dt}}\right)}_{\mathbf{e}}=\mathbf{N}\eta {\left(\frac{\mathbf{dV}}{\mathbf{dt}}\right)}_{\mathbf{single}} $$

Concluding, we use equations (7) and (2) to estimate the evaporation rate through our porous membrane.

### Internal flow rate

Next, the flow rate inside the rectangular microchannel will be examined. For a fully developed laminar flow in a rectangular channel the velocity profile reads (Papanastasiou et al. 1999)

8 $$ {\mathbf{u}}_{\mathbf{x}}\left(\mathbf{y},\mathbf{z}\right)=-\frac{1}{2\mu}\frac{\partial \mathbf{p}}{\partial \mathbf{x}}{\mathbf{c}}^2\left[1-{\left(\frac{\mathbf{z}}{\mathbf{c}}\right)}^2+4{\displaystyle \sum_{\mathbf{k}=1}^{\infty }}\frac{{\left(-1\right)}^{\mathbf{k}}}{\alpha_{\mathbf{k}}^3}\frac{ \cosh \left(\frac{\alpha_{\mathbf{k}}\mathbf{y}}{\mathbf{c}}\right)}{ \cosh \left(\frac{\alpha_{\mathbf{k}}\mathbf{b}}{\mathbf{c}}\right)} \cos \left(\frac{\alpha_{\mathbf{k}}\mathbf{z}}{\mathbf{c}}\right)\right] $$

where

9 $$ {\alpha}_{\mathbf{k}}=\left(2\mathbf{k}-1\right)\frac{\pi }{2},\kern0.5em \mathbf{k}=1,2,\dots $$

and *μ* is the dynamic viscosity of the liquid and p is the pressure.

**Fig. 4:**
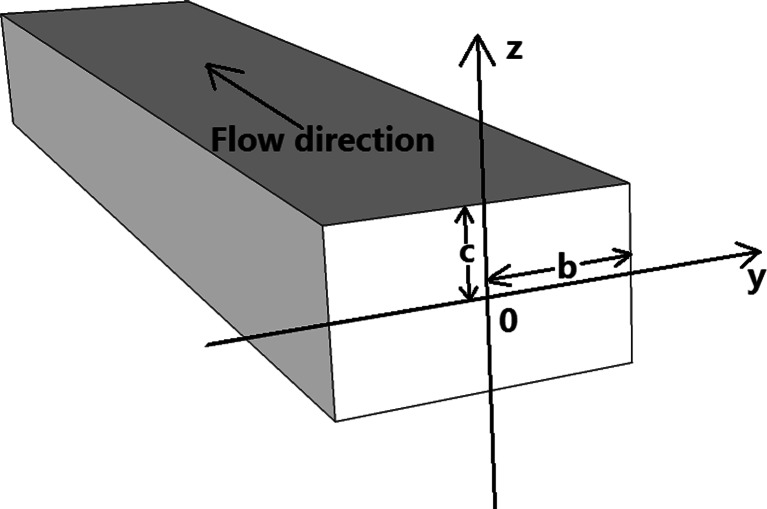
The geometrical channel parameters used for the 2D-PTV analysis

The x-coordinate is along the channel length, in the flow direction. We choose the mid-point of the rectangular cross-section channel as the origin (y,z) = (0,0). The geometrical parameters b and c are half of the width and half of the height of the cross-section respectively (see Fig. 4).

Then the volumetric flow rate Q can be calculated as (Papanastasiou et al. 1999):

10 $$ \mathbf{Q}=-\frac{4}{3\mu}\frac{\partial \mathbf{p}}{\partial \mathbf{x}}\mathbf{b}{\mathbf{c}}^3\left[1-\frac{6\mathbf{c}}{\mathbf{b}}{\displaystyle \sum_{\mathbf{k}=1}^{\infty }}\frac{ \tanh \left(\frac{\alpha_{\mathbf{k}}\mathbf{b}}{\mathbf{c}}\right)}{\alpha_{\mathbf{k}}^5}\right] $$

The pressure gradient $\frac{\partial \mathbf{p}}{\partial \mathbf{x}}$ in Eq. 10 is not directly measureable from the velocity profile distribution, but it can be estimated from Eq. 8 if the maximum velocity in the mid-point **u**_**x**_(0, 0), which can be measured directly, is known. Detailed calculations and equations are shown in the appendix.

By using Eqs. 1, 7 and 10, the measured flow rate within the channel can be compared with the estimated value from evaporation theory for an array of pores. According to Eq. 1, the two values should be equal.

## Results

### Liquid surface profile

The surface profile of the liquid–gas interface in a pore is the determined by the angle *θ* indicated in Fig. 3a. *θ* is needed to estimate the evaporation from a single pore, using Eqs. 2 and 3. The surface profile is measured with an optical profile meter (SENSOFAR microscope). A drawback of this measurement is that it cannot be done simultaneously with the flow recording experiments, and it is therefore done separately.

The profile of the liquid surface does not change as long as the liquid pressure in the channel is stable, i.e., it then remains in static equilibrium during pumping (θ’ is then constant during evaporation). During evaporation, the contact line remains pinned at the edge corner of the pores. The capillary pressure keeps transporting liquid from the channel to hold the pinned contact line.

**Fig. 5:**
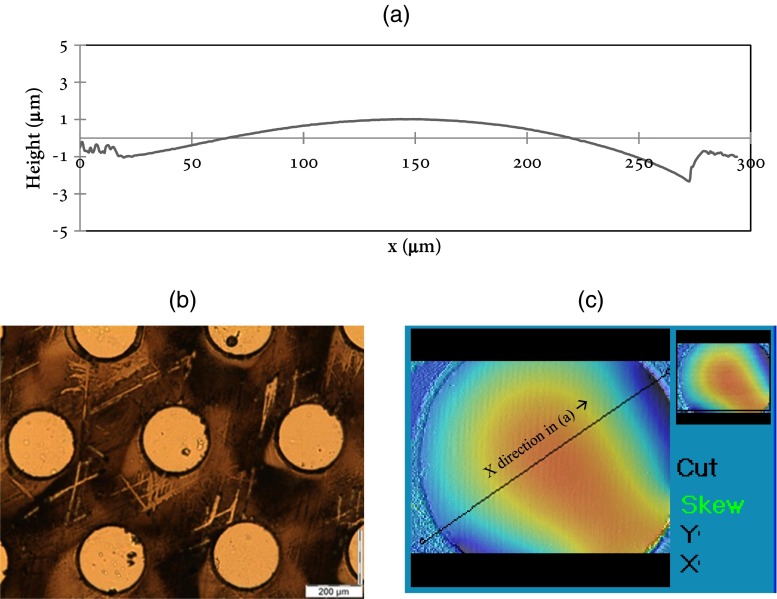
The liquid-air interface profile in a pore during evaporation pumping experiments, measured by interferometry. (**a**) liquid surface height profile across the center line of a pore; (**b**) Pores under the microscope; (**c**) one pore as characterized by an interferometry measurement; the profile in (**a**) is measured along the line shown in (**c**)

The circular pores in the porous structure have diameters in the range 50 μm to 250 μm. A typical result in Fig. 5 shows that the profile of the liquid–gas interface during the evaporation process is relatively flat, and the maximum height difference the measured liquid profile within the pore is around 3.5 μm which is much smaller than its diameter (D = 250 μm). From simple trigonometric functions the value of ***θ*** ' can be estimated as ***θ*** = 0.056 ***rad***. The resulting value of ***F***(***θ***) = 0.642 then follows from Eq. 3, which is close to (0.78 % larger than) the value for ***θ*** = 0 : ***F***(0) = 2/***π*** = 0.637. However, this value is not perfectly distributed evenly in one pore due to small imperfections caused by the manufacture process which is seen in Fig. 5a and c. For simplicity, the contact angle is approximated by ***θ*** = 0 in our estimations of the evaporation rate using the model presented in section 3.1.

### Evaporation and flow rate

**Fig. 6:**
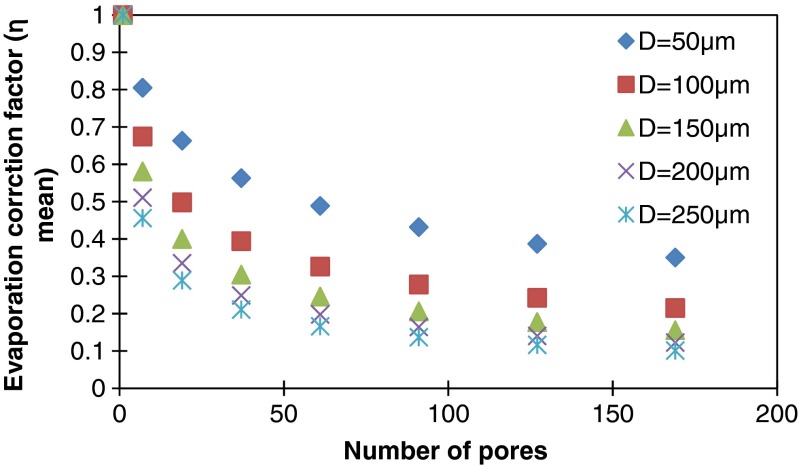
The evaporation correction factor vs. number of pores for different diameters D (μm) in a hexagonal array with a pitch of 500 μm

In the devices, the pores are distributed hexagonally. The evaporation correction factor is calculated through Equations 4 to 6. The pitch of the matrix of pores is set to 500 μm. Figure 6 shows the reduction of the mean evaporation correction factor when the number of pores increases. The evaporation correction factor also reduces for increasing pore size (while keeping the pitch constant) due to enhanced interaction between the pores. Although the evaporation flux per pore decreases with the number of pores, still the total evaporation rate increases slowly by adding more pores into the matrix because of the factor N in Equation 7.

**Fig. 7:**
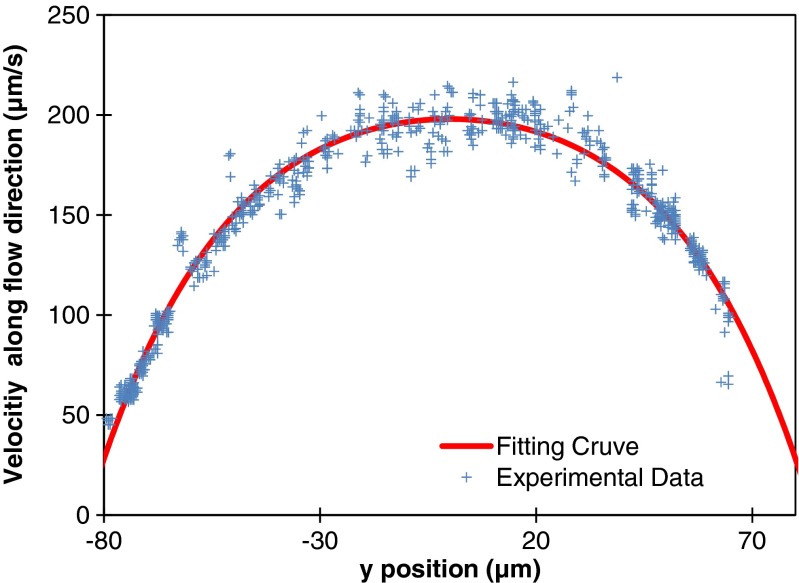
A typical result of measured velocity data using 2D PTV, fitted by Equation 8 using MATLAB. Experimental conditions: number of pores = 37, pore diameter = 200 μm, pitch = 500 μm, Tw = Ta = 23.1 °C, RH = 0.305, M = 1.8 × 10^-2^ kg/mol, D = 2.47 × 10^-5^ m^2^/s, ρ = 9.97 × 10^2^ kg/m^3^; fitted value: v_max_ = 198 μm/s

By using the 2D-PTV technique, focusing at the mid-plane of the channel, the speed of tracers in the liquid can be found by analyzing consecutive frames with a time gap *Δ* ***t***. The resulting velocity profile can be plotted as in Fig. 7, to which Equation 8 (z = 0) can be fitted to obtain the value for the maximum velocity.

The procedure to determine the flow rate was as follows (details can be found in the appendix). By processing all frames of the complete recordings of a single experiment, the experimental velocity profile (like in Fig. 7) was fitted by Eq. 8, z = 0. From this, the y-location of the maximum was determined, i.e., (y,z) = (0,0) was defined. Subsequently, the average and standard deviation of the data points located within the range of (y,z) = (0,0) ± (5 % of the channel width) were computed to find ***u***_***x***_(0, 0) and its experimental standard deviation. For the particular profile shown in Fig. 7, ***u***_***x***_(0, 0) =198 μm/s was found. Finally, this value of ***u***_***x***_(0, 0) was used to calculate the pressure gradient and subsequently the volumetric rate (and its standard deviation) using Eq. 10.

**Fig. 8:**
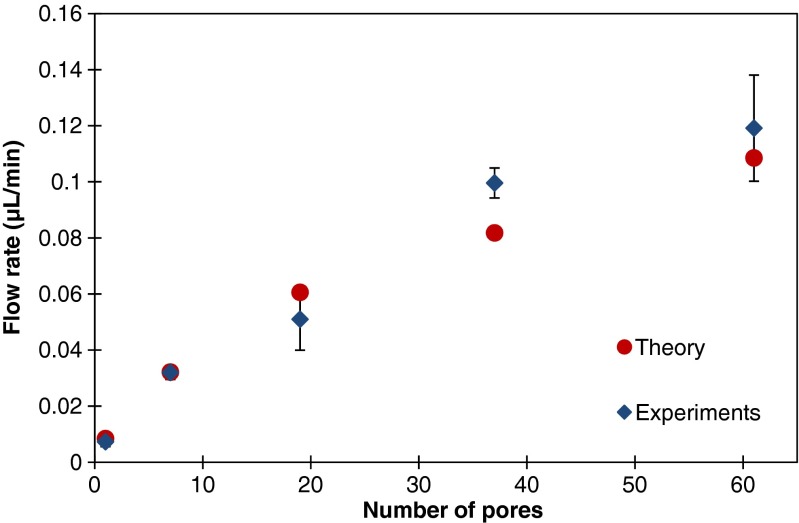
The dependency of flow rate on the number of pores. The pore diameter is fixed at 250 μm, with a pitch of 500 μm. The experimental values presented are the average and standard deviation values determined from ten recorded videos with duration of one minute. The theoretical values are calculated from evaporation theory

The results for the flow rate are shown in Fig. 8 as a function of the number of pores, along with the evaporation rate as estimated from evaporation theory (Eqs. 2 to 7). The experimentally measured and theoretically estimated flow rates are in fairly good agreement. The relationship between flow rate and the number of pores is not linear due to the reduction of the evaporation correction factor accounting for the interaction between neighboring pores. For this particular pore diameter (250 μm) and pitch (500 μm), a maximum flow rate of 0.12 μL/min is reached when the number of pores is 61.

**Fig. 9:**
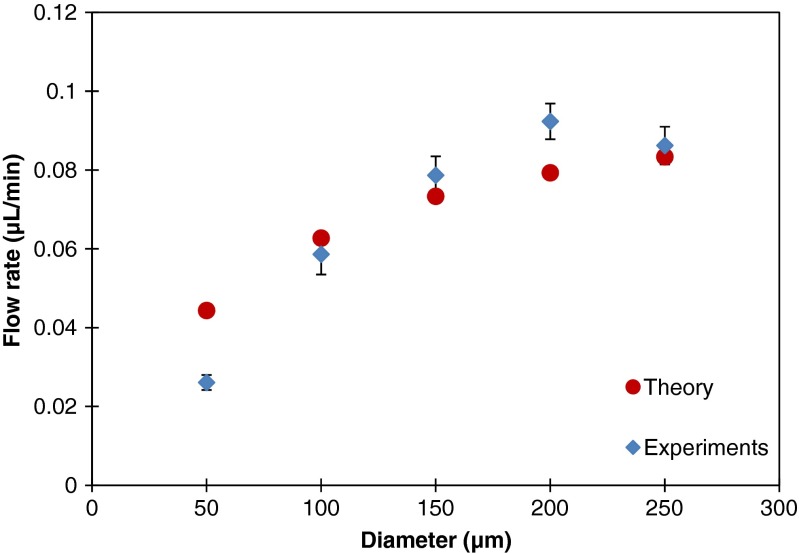
The flow rate as a function of pore diameter for an evaporation driven flow through an outlet with 37 pores with different pore diameters; the pitch between the pores is kept constant at 500 μm. The experimental values presented are the average and standard deviation values determined from ten recorded videos with duration of one minute. The theoretical values are calculated from evaporation theory

Furthermore, the influence of the diameter of the pores on the evaporation driven flow rate is studied. A set of pore diameters varying from 50 to 250 μm is used in the evaporation driven flow experiments. The theoretical values can again be estimated by Eqs. 2 to 7. The results are presented in Fig. 9. The flow rate increases when the diameter of the pores gets larger. The relation between the flow rate and diameter of pores is not linear, again due to the reduction of the evaporation correction factor. The experimental data show a good agreement with the theoretical values. Only for the smallest pore diameter, the results deviate. Most probably, this is due to the influence of the vertical pore wall, see Fig. 3a, which is 100 μm high in our measurements, and which becomes more important for smaller pore diameters. As explained in section 3.1, this effect is not accounted for in the theory.

When considering the application as a wearable sweat sensing system, the pump can be attached onto the human skin which is a natural temperature source. Therefore, the pumping behavior for different substrate temperatures is studied. To this end, a heater is applied as in Fig. 2. As explained before, in these experiments the heater does not extend underneath the inlet port in order to prevent extra evaporation at the inlet from influencing the measurement. In the complete device that represents the sweat sensor device (Fig. 1a) the upward facing inlet port is absent, so this additional evaporation is of no concern and the entire device would be heated when attached to the body’s surface.

**Fig. 10:**
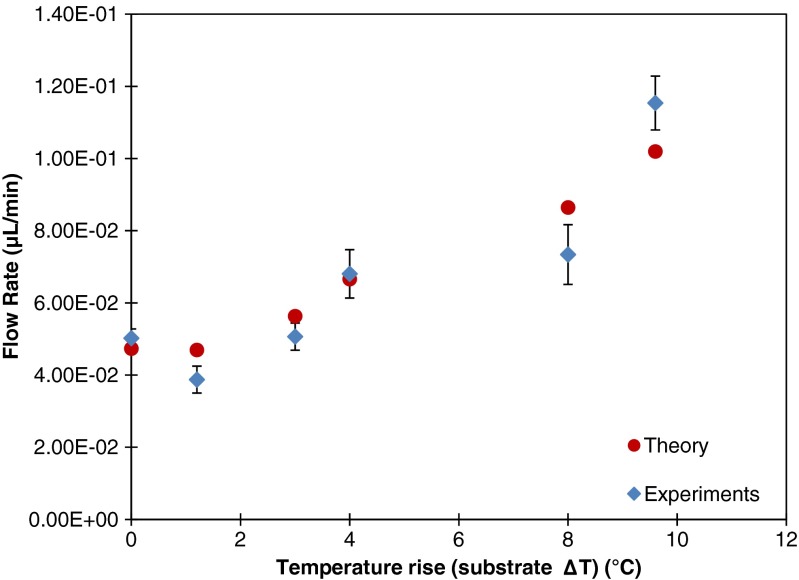
Flow rate measured for an outlet with 19 pores (diameter 250 μm, pitch 500 μm), as a function of the temperature rise at the outlet above the non-heated case (which is the reference). The experimental values presented are the average and standard deviation values determined from ten recorded videos with duration of one minute. The theoretical values are calculated from evaporation theory

An increase of the flow rate is observed with increasing temperature, as shown in Fig. 10. According to the evaporation theory given in section 3, the evaporation rate is sensitive to the water temperature through Eq. 2. We assume that the temperature rise is equal for the substrate and the liquid at the evaporation port. The water vapor concentration at the water-air interface is considered to be equal to the saturated concentration at the corresponding water temperature, which increases dramatically due to the heating of the liquid. The direct result of this change is a large increase of the vapor concentration difference. ***c***(***T***_***w***_) − ***Hc***(***T***_***a***_) Consequently, the evaporation rate and thus flow rate are increased. Figure 10 shows the results for the flow rate as a function of temperature rise (with respect to the non-heated reference, ***T***_***w***_ = ***T***_***a***_ = 21.4 ℃) in the case of an outlet with 19 pores (diameter =250 μm, pitch = 500 μm). A good agreement between the theoretical and experimental values is found.

### Fluid absorption and device filling

**Fig. 11:**
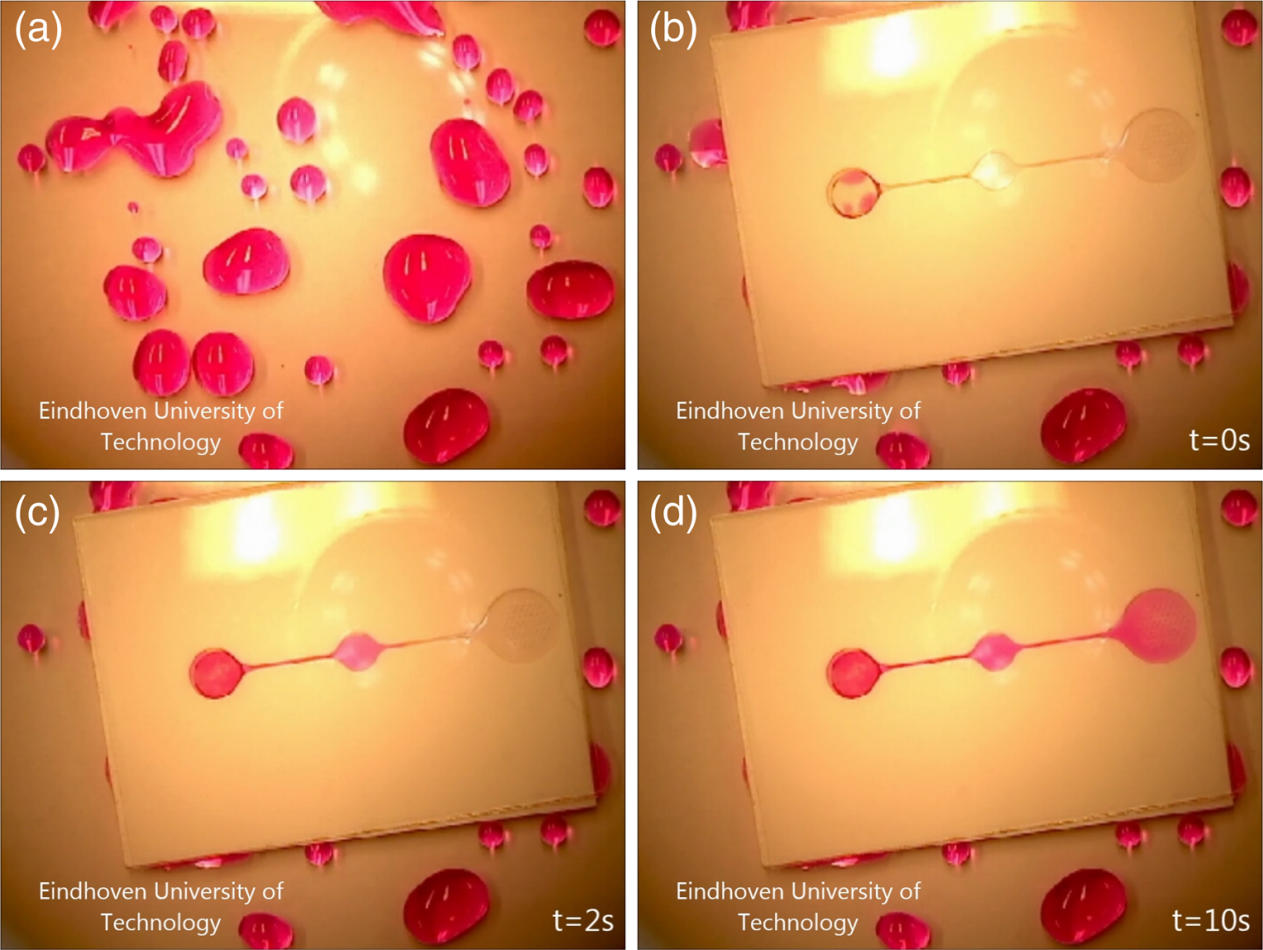
Colored water filling of an integrated system with a filter paper based interface layer, total time = 10 seconds. (**a**) Droplets on surface; (**b**) water is absorbed by the filter paper and transported to the inlet cavity; (**c**) water fills the sensing window in the middle of the system; (**d**) water reaches the outlet cavity and evaporation takes place. *Scale*: the size of the device shown is 40 by 30 mm. A movie of the process is shown in the Supplementary materials

Finally, the evaporative pump is connected to the fluid collection system as shown schematically in Fig. 1a. Figure 11 shows the results of the device. Drops of dyed fluid sitting on a surface are absorbed by the filter paper, the fluid is led to the channel inlet at the bottom of the device, and by capillarity the microchannel is filled up to the final cavity that contains the evaporation pore array. The whole process takes 10 seconds for this particular design. The process turned out to be robust, since all 5 samples made successfully functioned. A movie of the process is available in the supplementary materials. After fluid absorption and device filling evaporative pumping starts, as described in the previous section.

## Discussion

The experimental results of the flow rate are in fairly agreement with the values obtained from the evaporation theory except for the smallest pore diameter, and the measured trend follows the theory both in the passive and active (heated) modes. Hence the assumptions underlying the evaporation theory, listed in section 3.1, are fairly well obeyed except for the smallest pores for which, in the experiments, the vertical pore walls play a role that is not included in the model. In our experiments conducted in a laboratory environment, the environmental conditions such as temperature and humidity were controlled and monitored precisely. However, for the ultimate application of the sweat sensor, these conditions may vary and in practice may influence the evaporation speed and therefore the flow rate.

The ambient relative humidity (RH) values and air temperature (T) during our experiments are monitored by the RH/T sensor. The saturated vapor concentration increases dramatically when the temperature of the vapor increases. For instance, around a laboratory temperature of 20 °C, the saturated vapor pressure of water has a sensitivity larger than 5 %/°C (Hardy et al. 1998). The increase of saturated vapor pressure of water is even stronger at higher temperatures. The vapor concentration is calculated by multiplying the RH with the saturated vapor concentration at the measured temperature. That means that for a measurement tolerance of ±1 °C, the estimated evaporation rate and flow rate may vary between 94 % (at 19 °C) and 106 % (at 21 °C) of the value at 20 °C and RH = 40 % according to the concentration difference in Equation 2. The influence of humidity can be directly found in Eq. 2. When the liquid and ambient have the same temperature, the term ***c***(***T***_***w***_) = ***c***(***T***_***a***_) in Equation 2, then the evaporation rate is proportional to the value of (1 − ***H***). In the case of a disposable sweat sensor, the heat source may be the temperature of the skin itself, which ranges typically between 28 and 37 °C on forehead skin, in different situations (Boutcher et al. 1995). The variability in flow rate is large in this temperature range. In the case of 37 °C it could be 80 % higher than for the low temperature case (28 °C) at a room temperature of 20 °C and RH of 0.4. For some sweat sensing applications, such variability may be acceptable. In other cases however, a temperature monitoring system, such as an integrated thermal couple, is necessary to account for the variations and have better control over the flow rate.

The flow rate of our evaporation-driven pump can be controlled by varying the number, size, and pitch of the pores at the evaporation end from 7.3 × 10^-3^ to 1.2 × 10^-1^ μL/min for our particular designs. The sweating rate for people during exercise was studied in reference (Smith and Havenith 2011) and the variation is large between individual people and for different locations on the skin. The mean sweating rate mentioned by Smith and Havenith is 458 g/(m^2^h) which equals 0.763 μL/(cm^2^min) when we assume that the density of sweat equals 1000 kg/m^3^, and this rate can certainly be matched by our device when using multiple evaporation ends, or by resizing of the absorption paper area. The range of achievable flow rates can also be extended by controlling temperature. By increasing the temperature of the liquid, the flow rate is enhanced by about 130 % at a temperature rise of 9.4 °C.

An issue that is not addressed by our experiments is the potential effect of the accumulation at the pores of salt and other ions or biomolecules from the sweat. Over a longer time span, this may have a substantial effect on the evaporation process, and ultimately even block the pores, and this should be the subject of future efforts in developing to a full sweat sensor device.

As a final note, in the evaporation pumping experiments in which the simplified device shown in Fig. 1b was used, the evaporation happens at both inlet and outlet. At the inlet side, an even higher evaporation rate could in principle take place due to the larger liquid surface area compared to that in the porous outlet. One might argue that this could result in a flow from the outlet towards the inlet, however this does not happen since at the outlet, the individual liquid menisci are pinned at the pore edges, such that liquid flow towards the outlet must take place to obtain mass balance. To avoid a significant effect of the liquid meniscus at the inlet, liquid was added continuously to the inlet to prevent it from drying out. It is needed to keep the meniscus as flat as possible to reduce the pressure caused by the surface tension difference between the in- and outlet. If any, the surface tension difference between the inlet and outlet will be a temporary process which takes place immediately after the filling process, and thereafter will lead to only local flow near the inlet meniscus. The influence of evaporation at the inlet will be absent in the full sweat sensing device depicted in Fig. 1a, since this device has no opening to the ambient at the inlet but an interface with the skin for sweat collecting.

## Conclusions

We have provided proof-of-principle of a flexible microfluidic device that (1) automatically absorbs liquid through a filter paper interface, (2) fills the microfluidic features through capillarity, and (3) provides continuous fluid pumping through an evaporation micropump without requiring an additional power source. These properties make the device attractive for application as a wearable sweat sensing device. The evaporation driven micropump is realized on polymer sheets with as a key element a micro-porous membrane at the outlet. Its performance was predicted theoretically and evaluated experimentally. The fabrication process using laser machining is flexible and straightforward, and no clean room process is required for the fabrication procedures.

In our evaporation driven pump, the pumping performance shows good agreement with the evaporation behavior through an array of pores as predicted from evaporation theory. A maximum flow rate of 1.45 × 10^-1^ μL/min is reached in our experiments for a pore array with 61 pores (diameter = 250 μm, pitch = 500 μm) under normal laboratory conditions (Temperature: ~20 °C, RH: ~40 %) without forced air flow at the liquid–gas interface. This is well within the range of flow rates obtained by previously reported evaporative micropumps, as can be seen from Table 1. The flow rate can be controlled by varying the number, size, pitch of the pores, design and number of the evaporation outlets, or increasing the temperature. By a proper design, our micropump can certainly match the mean sweating rate of the human skin. For ultimate application as a sweat sensor, the heat from the body offers a “free” heat source that enhances the flow rate. Alternatively, a heating element can be integrated allowing for active control of the pumping rate.

### Electronic supplementary material

Below is the link to the electronic supplementary material.

ESM 1 (MPG 6434 kb)

## Appendix

### Water vapor concentration

From ideal gas law, the saturated vapor concentration obeys the following relationship:

A.1 $$ \boldsymbol{c}\left(\boldsymbol{T}\right)=\frac{\boldsymbol{n}}{\boldsymbol{V}}=\frac{{\boldsymbol{P}}_{\boldsymbol{s}}}{\boldsymbol{RT}} $$

where ***P***_***s***_ is the saturated vapor pressure at gas temperature, *T*, *R* is the gas constant, ***n*** is the amount of substance of vapor molecules in the volume ***V***. The saturated vapor pressure is approximated by (Current 2011)

A.2 $$ {\boldsymbol{P}}_{\boldsymbol{s}}=\boldsymbol{E}\boldsymbol{X}\boldsymbol{P}\ \left(20.386-\frac{5132}{\boldsymbol{T}}\right)\boldsymbol{mmHg} $$

### Calculation of flow rate in a rectangular channel

From equation 8 follows that the maximum velocity u_x_(0,0) can be written as:

A.3 $$ {\boldsymbol{u}}_{\boldsymbol{x}}\left(0,0\right)=-\frac{1}{2\boldsymbol{\mu}}\frac{\partial \boldsymbol{p}}{\partial \boldsymbol{x}}{\boldsymbol{c}}^2\left[1+4{\displaystyle \sum_{\boldsymbol{k}=1}^{\infty }}\frac{{\left(-1\right)}^{\mathbf{k}}}{ \cosh \left(\frac{\upalpha_{\mathrm{k}}\mathrm{b}}{\mathrm{c}}\right){\upalpha}_{\mathrm{k}}^3}\right] $$

where

$$ {\upalpha}_{\mathrm{k}}=\left(2\mathrm{k}-1\right)\frac{\uppi}{2},\kern0.5em \mathrm{k}=1,2,\dots $$

From equation (A.3) the pressure gradient $\frac{\partial \mathrm{p}}{\partial \mathrm{x}}$ can be calculated:

A.4 $$ \frac{\partial \mathrm{p}}{\partial \mathrm{x}}=-\frac{2{\upmu \mathrm{u}}_{\mathrm{x}}\left(0,0\right)}{{\mathrm{c}}^2}{\left\{\frac{32\left[{\displaystyle {\sum}_{\mathrm{k}=1}^{\infty }}\frac{2{\left(-1\right)}^{\mathrm{k}}}{\left(\mathrm{E}\mathrm{X}\mathrm{P}\left(-\frac{\mathrm{b}\uppi \left(2\mathrm{k}-1\right)}{2\mathrm{c}}\right)+\mathrm{E}\mathrm{X}\mathrm{P}\left(\frac{\mathrm{b}\uppi \left(2\mathrm{k}-1\right)}{2\mathrm{c}}\right)\right)}\right]}{\uppi^3}+1\right\}}^{-1} $$

Also the velocity at the midplane u_x_(y, 0) can be written as a function of maximum velocity u_x_(0, 0)

A.5 $$ {\mathrm{u}}_{\mathrm{x}}\left(\mathrm{y},0\right)={\mathrm{u}}_{\mathrm{x}}\left(0,0\right)\frac{32{\displaystyle {\sum}_{\mathrm{k}=1}^{\infty }}\frac{{\left(-1\right)}^{\mathrm{k}}\left(\frac{1}{2{\upsigma}_2}+\frac{\upsigma_2}{2}\right)}{\upsigma_1{\upsigma}_3}+{\uppi}^3}{32{\displaystyle {\sum}_{\mathrm{k}=1}^{\infty }}\frac{{\left(-1\right)}^{\mathrm{k}}}{\upsigma_1{\upsigma}_3}+{\uppi}^3} $$

where

$$ \begin{array}{c}\hfill {\upsigma}_1=\frac{1}{2}\left({\mathrm{e}}^{-\frac{\mathrm{b}\uppi \left(2\mathrm{k}-1\right)}{2\mathrm{c}}}+{\mathrm{e}}^{\frac{\mathrm{b}\uppi \left(2\mathrm{k}-1\right)}{2\mathrm{c}}}\right)\hfill \\ {}\hfill {\upsigma}_2={\mathrm{e}}^{\frac{\mathrm{y}\uppi \left(2\mathrm{k}-1\right)}{2\mathrm{c}}}\hfill \\ {}\hfill {\upsigma}_3={\left(2\mathrm{k}-1\right)}^3\hfill \end{array} $$

We chose to let k run from 1 to 50 to fit the measured velocity profile and find the location of the maximum, i.e., the midpoint . The maximum velocity u_x_(0, 0) was then determined by calculating the average of all measured data points within a distance from the midpoint of 5 % of the channel width. With this, $\frac{\partial \mathrm{p}}{\partial \mathrm{x}}$ and the volumetric rate Q were calculated as described in the main text.
